# THE IMPACT OF DEPRESSION ON OBJECTIVE AND SUBJECTIVE ORAL HEALTH INDICATORS IN OLDER INDIVIDUALS

**DOI:** 10.1093/geroni/igae098.4087

**Published:** 2024-12-31

**Authors:** Boyun Choi, Bock-Young Jung

**Affiliations:** Yonsei University, Seoul, Republic of Korea; Yonsei University College of Dentistry, Seoul, Republic of Korea

## Abstract

Depression significantly affects overall health and quality of life, including oral health and subjective discomfort. This study examines how objective and subjective oral health factors differ based on depression diagnosis in individuals aged 65 and older. Using data from the 2018-2020 National Health and Nutrition Survey (n=3324) by the Korea Centers for Disease Control and Prevention, this study compared oral health in patients with and without depression. The research assessed subjective factors like chewing difficulties, self-perceived oral health, and recent tooth pain, alongside objective factors such as the number of natural teeth and the need for prosthetics. Analyses included Rao-Scott chi-square tests, survey logistic regression, and linear regression. Significant differences were observed in subjective oral health factors between the depressed and non-depressed groups. Depression was associated with recent tooth pain (p = 0.0022), chewing discomfort (p < 0.0001), and self-perceived poor oral health (p = 0.0136). Regardless of gender, age, BMI, depression group reported the same pattern. However, no significant differences were observed in objective factors such as the number of natural teeth or the need for prosthetics. These findings suggest that depression could influence oral subjective and functional ability factors more sensitively and how older individuals perceive their oral health.

